# Definitive Radiochemotherapie von lokal fortgeschrittenen Kopf-Hals-Tumoren in Kombination mit Immuncheckpointinhibition – neue Konzepte gefragt

**DOI:** 10.1007/s00066-021-01880-4

**Published:** 2021-12-06

**Authors:** Markus Hecht, Rainer Fietkau, Udo S. Gaipl

**Affiliations:** Strahlenklinik, Friedrich-Alexander-Universität Erlangen-Nürnberg, Universitätsstr. 27, 91054 Erlangen, Deutschland

## Hintergrund und Ziele

Immuncheckpointinhibitoren gegen den PD‑1(„programmed cell death protein 1“)/PD-L1(„programmed cell death ligand 1“)-Signalweg sind zur Therapie von rezidivierten und oder metastasierten Kopf-Hals-Tumoren zugelassen. Pembrolizumab wird aktuell in diesen Stadien als Erstlinientherapie entweder als Monotherapie oder in Kombination mit Chemotherapie (ChT) eingesetzt [1]. Bei inoperablen, lokal fortgeschrittenen Kopf-Hals-Tumoren ist die definitive Radiochemotherapie (RCT) mit drei Kursen Cisplatin in Hochdosis (100 mg/m^2^ Körperoberfläche) die Therapie der Wahl. Zur Kombination von Strahlentherapie (RT) und Immuncheckpointinhibitoren existieren vielversprechende präklinische Daten, da durch die immunmodulatorischen Effekte einer RT eine zusätzliche Immunaktivierung erwartet wird [2]. In der hier zu kommentierenden randomisierten, doppelt verblindeten internationalen Phase-III-Studie (Javelin-Head-and-Neck-100-Studie) wird der Einsatz des PD-L1-Inhibitors Avelumab in Kombination mit einer definitiven RCT bei lokal fortgeschrittenen Kopf-Hals-Tumoren untersucht.

## Patienten und Methoden

Patienten mit Erstdiagnose eines lokal fortgeschrittenen Plattenepithelkarzinoms der Mundhöhle, des Oropharynx, des Hypopharynx oder des Larynx konnten, wenn sie ohne Fernmetastasen waren, an dieser Studie teilnehmen. Eine biomarkerbasierte Patientenselektion erfolgte nicht. Alle Patienten erhielten eine definitive RCT bis 70 Gy in 35 Fraktionen, appliziert als intensitätsmodulierte Radiotherapie (IMRT). Die simultane Chemotherapie erfolgte mit Cisplatin (100 mg/m^2^ KO, 3 Kurse, appliziert alle 3 Wochen). Es erfolgte eine Randomisierung (1:1) auf eine zur RT simultane Therapie mit dem PD-L1-Inhibitor Avelumab (10 mg/kg KO, appliziert alle 2 Wochen), gefolgt von einer Erhaltungstherapie über 12 Monate oder zur Kontrolle Placebo. Primärer Endpunkt war das progressionsfreie Überleben (PFS).

## Ergebnisse

Zwischen Dezember 2016 und Januar 2019 wurden 697 Patienten randomisiert. Die relevanten Prognoseparameter waren in beiden Gruppen gleich verteilt. Abbrüche der Strahlentherapie wurden in der Avelumabgruppe bei 23 (von 345) Patienten und in der Placebogruppe bei 20 (von 340) Patienten registriert. Die mediane Cisplatindosis betrug 260 mg/m^2^ in der Avelumabgruppe und 278 mg/m^2^ in der Placebogruppe. Die mediane Nachbeobachtungszeit für das PFS betrug 14,6 Monate in der Avelumabgruppe und 14,8 Monate in der Placebogruppe. Beim primären Endpunkt PFS ergab sich ein leichter Trend zugunsten der Placebogruppe (Hazard Ratio 1,21; 95 %-Konfidenzintervall 0,93–1,57; *p* = 0,92). Hinsichtlich des Gesamtüberlebens (OS) waren die Unterschiede geringer, aber ebenfalls zugunsten der Placebogruppe. Nebenwirkungen ≥ Grad 3 traten bei 88 % der Patienten in der Avelumabgruppe und bei 82 % der Patienten in der Placebogruppe auf. Immunassoziierte Nebenwirkungen ≥ Grad 3 traten bei 5 % der Patienten in der Avelumabgruppe und bei 2 % der Patienten in der Placebogruppe auf. In den explorativen Analysen erstreckte sich dieser negative Trend der Avelumabgruppe über sämtliche Subgruppen. Einzig Patienten mit PD-L1 hoch exprimierenden Tumoren (≥ 25 %) schienen von der Avelumabgabe zu profitieren (Hazard Ratio 0,59; 95 %-Konfidenzintervall 0,28–1,22).

## Schlussfolgerung der Autoren

Eine Verlängerung des progressionsfreien Überlebens konnte durch die Hinzunahme von Avelumab zur definitiven RCT nicht erreicht werden. Dennoch können die Erfahrungen mit dieser Studie dazu beitragen, das Design zukünftiger Studien zur Kombination von Immuncheckpointinhibitoren und RCT zu verbessern.

## Kommentar

Die platinbasierte definitive RCT ist die Standardbehandlung für lokal fortgeschrittene inoperable Kopf-Hals-Tumoren. In der letzten deutschen Phase-III-Studie konnte mit dieser Therapie nach 3 Jahren ein PFS von 58 % und ein OS von 65 % erreicht werden [3]. Die lokoregionäre Rezidivrate betrug 18 % und die Fernmetastasierungsrate 10 %. Dies sind zwar gute Ergebnisse für inoperable Kopf-Hals-Tumoren, es wird aber auch klar, dass noch Verbesserungsbedarf besteht. Durch die Einführung der Immuncheckpointinhibitoren kam eine neue Therapieoption für Kopf-Hals-Tumoren ins Spiel. In der rezidivierten und/oder metastasierten Situation ist eine Immuntherapie mit dem PD-1-Inhibitor Pembrolizumab (allein oder in Kombination mit ChT) bereits Erstlinientherapie [1]. Hieraus entstand das Studienkonzept einer Kombination von definitiver RCT mit simultaner PD-1/PD-L1-Immuncheckpointinhibition für lokal fortgeschrittene Kopf-Hals-Tumoren. Die Kombination von RT und Immuntherapie wird mittlerweile von soliden präklinischen Daten gestützt, die u. a. eine Verbesserung der lokalen und systemischen Tumorkontrolle durch die immunmodulatorischen Effekte einer Strahlentherapie zeigen [2]. So war das untersuchte Therapiekonzept in der Javelin-Head-and-Neck-100-Studie aus wissenschaftlicher Sicht durchaus erfolgsversprechend.

Die Javelin-Head-and-Neck-100-Studie erfüllte alle Qualitätskriterien einer Phase-III-Studie. Die Studie war randomisiert, placebokontrolliert und doppelt verblindet. Es gab eine zentrale Qualitätskontrolle für Bestrahlungspläne. Insgesamt war die Studie eindeutig negativ bezüglich des primären Endpunkts PFS. Lediglich in der Subgruppe der Patienten mit PD-L1 hoch exprimierenden Tumoren (PD-L1 ≥ 25 %) schienen die Patienten vom PD-L1-Inhibitor zu profitieren. Dies zeigt klar die Notwendigkeit der Patientenselektion. In der metastasierten und/oder rezidivierten Situation ist die Ansprechrate auf PD-1-Inhibitoren umso höher, je höher PD-L1 auf Tumor- und Immunzellen exprimiert wird [1]. Neben PD-L1 könnten die Tumormutationslast (TMB), genetische Signaturen (z. B. γ‑Interferon) oder immunologische Blutparameter als prädiktive Parameter dienen [4]. Spannend wird noch das Ergebnis der Keynote-412-Studie, die im identischen Design den Nutzen des PD-1-Inhibitors Pembrolizumab in Kombination mit RCT untersucht. Ob die Blockade des PD-1/PD-L1-Signalwegs mit einem den Rezeptor hemmenden PD-1-Inhibitor in dieser Situation effektiver ist als mit einem gegen den Liganden gerichteten PD-L1-Inhibitor, bleibt abzuwarten.

Insgesamt zeigt dieses Studienergebnis deutlich, dass die Hinzunahme von Immuncheckpointinhibitoren zur definitiven RCT ohne eine Patientenselektion oder Anpassung des strahlentherapeutischen Konzepts nicht zum Erfolg führt. Daher sollten neue Konzepte überlegt werden. Eine vielversprechende Möglichkeit zeigte kürzlich das Konzept der CheckRad-CD8-Studie, in der nach einem Zyklus Induktionschemoimmuntherapie Patienten anhand intratumoraler CD8+-Immunzellen für eine chemotherapiefreie Radioimmuntherapie selektioniert wurden [5]. In dieser Studie erfolgte die Immuncheckpointinhibition allerdings kombiniert mit PD-L1- und CTLA-4-Inhibitor.

Eine komplett chemotherapiefreie Radioimmuntherapie ohne vorherige Patientenselektion wurde in der REACH-Studie untersucht. Hier war die Studientherapie mit dem PD-L1-Inhibitor in Kombination mit Cetuximab simultan zur Strahlentherapie einer platinbasierten RCT unterlegen, was die Notwendigkeit der Chemotherapie als Induktion und/oder simultan zur Bestrahlung unterstreicht [6]. Eine Weiterentwicklung der Systemtherapie stellt der Antagonist der Inhibitor-of-apopsisis-Proteine (IAP) Xevinapant dar (früher: Debio-1143). In einer randomisierten Phase-II-Studie konnte Xevinapant simultan zur platinbasierten RCT das PFS signifikant verbessern [7]. Möglicherweise müssen in Kombination mit Immuntherapie auch etablierte Bestrahlungskonzepte überdacht werden. Für die Immunreaktion ist die Ausreifung der Immunzellen in regionären Lymphknoten ein wichtiger Schritt. Präklinische Untersuchungen konnten nämlich zeigen, dass im Tiermodell bei gleichzeitiger Bestrahlung und Immuncheckpointinhibition die Immunreaktion und Tumorkontrolle signifikant vermindert sind, wenn die regionären Lymphknoten mitbestrahlt werden [8]. Daher sollten auch strahlentherapeutische Behandlungskonzepte für künftige Studien mit kombinierter Radioimmuntherapie überdacht werden.

## Fazit

Durch die Hinzunahme des PD-L1(„programmed cell death ligand 1“)-Inhibitors Avelumab zur definitiven Radiochemotherapie konnte im Gesamtkollektiv zwar keine Verlängerung des progressionsfreien Überlebens erreicht werden, doch scheinen Patienten mit PD-L1 hoch exprimierenden Tumoren von der PD-L1-Inhibition zu profitieren. Bei der Planung zukünftiger Studien sollte neben einer Patientenselektion auch über Induktionstherapien und alternative Bestrahlungskonzepte nachgedacht werden.

*Markus Hecht, Rainer Fietkau und*

*Udo S. Gaipl, Erlangen*

## Interessenkonflikt

M. Hecht: Merck Serono (Advisory Boards, Vorträge, Reisemittel, Forschungsfinanzierung); MSD (Advisory Boards, Vorträge, Reisemittel, Forschungsfinanzierung); AstraZeneca (Forschungsfinanzierung); Novartis (Forschungsfinanzierung); BMS (Advisory Boards, Vorträge); Teva (Reisemittel). R. Fietkau: MSD (Advisory Boards, Reisemittel, Forschungsfinanzierung); Fresenius (Sonstiges); BrainLab (Sonstiges); AstraZeneca (Advisory Boards, Reisemittel, Forschungsfinanzierung); Merck Serono (Advisory Boards, Reisemittel, Forschungsfinanzierung); Novocure (Advisory Boards, Vorträge, Forschungsfinanzierung); Sennewald (Vorträge, Reisemittel). U.S. Gaipl: AstraZeneca (Advisory Boards, Forschungsfinanzierung); BMS (Advisory Boards); MSD (Forschungsfinanzierung); Sennewald Medizintechnik (Reisemittel, Advisory Boards).
